# INFLUENCING FACTORS OF SUBJECTIVE AGE: FINDINGS FROM THE KUSATSU LONGITUDINAL STUDY ON AGING AND HEALTH

**DOI:** 10.1093/geroni/igz038.2561

**Published:** 2019-11-08

**Authors:** Tomoko Ikeuchi, Satoshi Seino, Yu Taniguchi, Miki Narita, Takumi Abe, Hidenori Amano, Akihiko Kitamura, Shoji Shinkai

**Affiliations:** Tokyo Metropolitan Institute of Gerontology, Tokyo, Japan

## Abstract

Background: Subjective age (SA) has been found to be a biopsychosocial marker of aging, yet little is known about factors that influence SA development. This study examined factors influencing SA using longitudinal data of community-dwelling older Japanese. Methods: Data drawn from the Kusatsu Longitudinal Study were collected during annual health check-ups in 2017 and 2018 from participants (aged 65-95) who completed all the measurement items used for this analysis (N=981). SA was indexed by asking participants to specify in years how old they felt. Proportional discrepancy scores ((subjective age - chronological age)/chronological age ×100) were calculated to indicate younger or older SAs and used as a dependent variable. As influencing factors of SA, chronological age, sex, years of schooling, history of smoking, cognitive function (using MMSE scores, range 14-30 at baseline), depressive symptoms, physical function (gait speed), and social function (employment status) were examined. Analyses were performed with random-effects GLS regression models. Results: Significant partial regression coefficients were found for cognitive function (0.48%, CI: 0.18, 0.79), years of schooling (-0.42%, CI: -0.69, -0.15), depressive symptoms (0.32%, CI: 0.11, 0.53), and chronological age (-0.18%, CI: -0.30, -0.68). Implications: This study found that older age and longer years of schooling were associated with younger SA, while better cognition and depressive symptoms were linked to older SA. Better cognition being associated with older SA was inconsistent with existing studies. This may be due in part to the association of better cognition and the level of satisfaction influenced by awareness of age-related physical/social changes.

